# Dr. Gibbes on the Melksham Water

**Published:** 1813-11

**Authors:** G. S. Gibbes

**Affiliations:** Bath


### *To the Editor of the Medical and Physical Journal.*

SIR,

AS I have repeated my experiments several times on the Melksham water, and as I wish to make the account you honored me by inserting in your valuable Journal, as correct as possible, I beg leave to submit to you this short statement of the analysis. I also send you the results of experiments made for the purpose of establishing the real contents of the Cheltenham and Leamington waters, being authorised by a medical friend, who has devoted much time to the processes by which they were ascertained, the detail of which will shortly be presented by him to the public. You will observe that these analyses differ from such as have been lately given of these celebrated springs; and you will also see that the salts here enumerated are not incompatible with each other.

A quantity of the residuum which had been obtained from evaporating the Melksham water at the heat of 180°, was subjected to alcohol, and repeatedly washed in it until all the earthy muriates were dissolved and carried off. To this filtered solution both carbonate of ammonia and pure ammonia were added, and each produced a precipitation evincing the presence of both muriate of lime and muriate of magnesia.

Distilled water was then poured on the residuum to examine the nature of the sulphates, but nitrate of lime produced no precipitation in this filtered liquor, and pure ammonia did not detect any magnesia, consequently neither sulphate of soda nor sulphate of magnesia exist in this water.

The remaining residuum was dissolved in dilute nitric acid, and the fluid was filtered, leaving the sulphate of lime on the filter. In this fluid pure ammonia produced a slight precipitation showing magnesia; carbonate of ammonia separated the calcareous earth; and prussiate of potash evinced, by a strong blue color, the presence of iron.

The contents of this water, therefore, are muriate of soda, muriate of magnesia, muriate of lime, sulphate of lime, and the the carbonates of lime, magnesia, and iron. I have put down the carbonate of magnesia, which, though questionable as an ingredient existing with muriate of lime, my experiments have uniformly detected. The muriate of lime being incompatible with the sulphates, except sulphate of lime, we could not expect it to exist in the Cheltenham waters, where Glauber's salt is a well-known ingredient.

The waters of Mr. Thompson's chalybeate and carbonate saline wells, as ascertained by the gentleman above mentioned, contain muriate of soda and sulphate of soda, no muriate of lime, no muriate of magnesia, and no sulphate of lime; a small portion of carbonate of soda, and, perhaps, a grain or two in a pint of sulphate of magnesia; the carbonates of lime, magnesia, and iron.

The water of Forty's old well contains muriate of soda, sulphate of soda, muriate of magnesia, sulphate of magnesia, and sulphate of lime; the three carbonates of lime, magnesia, and iron.

Leamington water contains muriate of soda, sulphate of soda, muriate of magnesia, a small quantity of sulphate of magnesia, and a very large portion of sulphate of lime.

From the above statement, it appears that the peculiarity of the Melksham water consists in its containing muriate of lime, and in its possessing at the same time a purgative quality, from which it may be entitled to some medical respect, and may establish some degree of reputation similar to that which has been so deservedly gained by the celebrated waters of Cheltenham and Leamington.

The Melksham water has been for some time used by the common people with much success for glandular, scrophulous, and scorbutic complaints; and already many very decided cases of those disorders, as well as such as proceed from indigestion and improper secretion of bile, have come to my knowledge. I may take the liberty of sending to you, at some future time, the detail of cases, as there seems sufficient reason to suspect that this variety of mineral water may have a correspondent effect on disease.

I have the honor to be, Sir,

Your most obedient Servant,

*Bath, Oct.* 10, 1813. G. S. GIBBES, M.D.

P. S.—I have not mentioned the quantities of each ingredient contained in the Melksham water, as I wait to compare the results of my experiments with those of Dr. Wilkinson, who is now engaged in the analysis.

